# Rapid detection of carbapenemase activity of *Enterobacteriaceae* isolated from positive blood cultures by MALDI-TOF MS

**DOI:** 10.1186/s12941-018-0274-9

**Published:** 2018-05-18

**Authors:** Jiajia Yu, Jingxian Liu, Yuanrui Li, Jing Yu, Weinan Zhu, Ying Liu, Lisong Shen

**Affiliations:** Department of Clinical Laboratory, Xin Hua Hospital, Shanghai Jiao Tong University School of Medicine, 1665 Kong Jiang Road, Yangpu District, Shanghai, 200092 China

**Keywords:** MALDI-TOF MS, Blood culture, Carbapenemase, *Enterobacteriaceae*, Hydrolysis

## Abstract

**Background:**

Matrix-assisted laser desorption ionization-time of flight mass spectrometry (MALDI-TOF MS) has been proved to be a useful tool for identification of pathogens directly isolated from blood cultures in clinical microbiology laboratories. β-Lactam antibiotics are commonly used for treatment of bloodstream infections caused by *Enterobacteriaceae* strains, and carbapenem is the superlative class of β-lactam antibiotics. Since the carbapenem resistance rate of *Enterobacteriaceae* strains raised year by year, efficient detection of carbapenemase activity and timely delivery of carbapenem susceptibility reports of *Enterobacteriaceae* strains isolated from blood cultures is important for clinicians.

**Methods:**

We used 64 simulated blood cultures to establish the method of MALDI-TOF MS based ertapenem hydrolysis assay. The cutoff value of logRQ calculated from the peaks intensity of ertapenem and its hydrolysate was first set to identify the strains with carbapenemase activity. Then, we detected and calculated the logRQ values of 385 *Enterobacteriaceae* strains from positive clinical blood cultures to distinguish the carbapenemase producers and noncarbapenemase producers.

**Results:**

The mean logRQ value of 32 noncarbapenemase producers was − 0.85 ± 0.14 in simulated blood cultures, while the logRQ value of 32 carbapenemase producers was 0.87 ± 0.55. Thus, the cutoff value of logRQ was set at − 0.45 with sensitivity of 100% and specificity of 100%. In 385 clinical positive blood cultures, the logRQ values of all carbapenem-susceptible *Enterobacteriaceae* strains (81.3%, 313/385) were < − 0.45. Comparing with the detection of carbapenemase genes, carbapenem-resistant *Enterobacteriaceae* strains (18.7%, 72/385) were well distinguished by MALDI-TOF MS based ertapenem hydrolysis assay with a sensitivity of 92.5% and specificity of 100%.

**Conclusions:**

Our data show that MALDI-TOF MS based ertapenem hydrolysis assay is a rapid and accurate method to detect carbapenemase activity of *Enterobacteriaceae* strains from positive blood cultures, and can be routinely performed in clinical microbiology laboratories.

## Background

Bloodstream infection is a severe threat for human life. As reported before, a mean survival rate of 7.6% was decreased if the effective treatment was applied per hour delay for patients in septic shock [1]. Therefore, early appropriate antibiotic treatment is important to improve prognosis of bloodstream infections [2]. Blood culture was considered as the ‘gold standard’ test for the detection of pathogens in blood specimens [3]. The rapid identification and susceptibility test reports for the pathogens in blood specimens are very meaningful for clinicians to choose appropriate and efficient antimicrobial agents for anti-infective therapy. Nowadays, *Enterobacteriaceae* strains are becoming the major pathogens which can cause bloodstream infections with an isolating rate up to 30% [4]. Carbapenem is the crucial antibacterial agent for primary empirical antibacterial therapy against infections caused by *Enterobacteriaceae* strains. However, In the past few years, along with the large-scale use of antibiotics, carbapenem resistance of *Enterobacteriaceae* strains has increased rapidly and become one of the most common global health issues [5], especially for the carbapenem resistance rate of clinical isolated *Klebsiella pneumoniae*, which has reached up to 10% in 2014 [6, 7]. In addition, empirical antibacterial monotherapy may not well inhibit the growth of resistant *Enterobacteriaceae* strains [8]. Therefore, it’s important for clinical microbiology laboratories to give clinicians timely and believable antimicrobial reports for early anti-infective treatment.

MALDI-TOF MS has been routinely used to identify microorganisms in clinical microbiology laboratories. Recently, it’s reported that MALDI-TOF MS has been applied to the antimicrobial resistance research, especially for the activity detection of hydrolytic enzyme that produced by microorganisms. Here, we evaluated the efficiency of MALDI-TOF MS for detection the carbapenemase hydrolyze activity of *Enterobacteriaceae* strains isolated from positive blood cultures.

## Methods

### Blood culture simulations

64 *Enterobacteriaceae* strains were collected from patient specimens in the clinical microbiology laboratory of Xinhua Hospital, Shanghai JiaoTong University School of Medicine, Shanghai, China. The strains were constitute of 32 carbapenemase producers and 32 noncarbapenemase producers, and were stored in glycerol broth at − 80 °C. For each strain, after be inoculating and growing on Columbia blood plate at 35 °C, bacterial suspensions of 0.5 McFarland were prepared with 0.45% NaCl, and then diluted 100 times by 0.45% NaCl. 200 μL of bacterial suspensions were injected into the negative blood culture bottles to simulate blood culture specimens. These blood culture bottles were incubated at 35 °C in the BD BACTEC FX Blood Culture System (Bectom Dickinson), and were taken out and detected by MALDI-TOF MS based ertapenem hydrolysis assay when the positive alerting signal was observed from the system.

### MALDI-TOF MS based ertapenem hydrolysis assay

4 mL samples from positive blood cultures were injected into serum separator tubes (ST740CG, INSEPACK^®^, Japan), and centrifuged at 3000 × *g* for 10 min in order to remove blood cells and debris at the bottom of tubes. The bacterial pellets at the surface of separation gel were carefully transferred into a 1.5 mL tube and washed twice using 500 μL 0.45% NaCl. Then the pellets were resuspended with 50 μL of ertapenem solution (0.2 mg mL^−1^ ertapenem, 20 mmol L^−1^ Tris–HCl, 0.45% NaCl) and then incubated at 35 °C for 2 h. The ertapenem solution without any bacteria was set as the blank control. After 2 h incubation, the suspensions were centrifuged at 3000 × *g* for 5 min. 1 μL of the supernatant was spotted on the target plate (Bruker Daltonics), subsequently mixed with 1 μL HCCA matrix solution (10 mg mL^−1^ cyano-4-hydroxycinnamic acid) and then dried at room temperature. Mass spectrum was acquired by FlexControl 3.0 software (Bruker Daltonics) of Microflex LT mass spectrometer (Bruker Daltonics) in the low mass-to-charge range (100–1000) with 60 Hz laser frequency and 200 laser shots. The matrix peaks (HCCA [M + H]^+^ at 190.05 and [2 M + H]^+^ at 379.02) were used for calibration per test. Further analysis of mass spectrum was performed by the Flexanalysis 3.0 (Bruker Daltonics). Firstly, the spectrum was smoothed and baseline was subtracted. Then, the specific ertapenem related peaks and hydrolysate related peaks were manually checked in the mass-to-charge range of 440–560.

### LogRQ values

The logRQ values which were used to estimate the results of hydrolysis assay were calculated by the intensities of peaks. The formula was logRQ = log (sum of hydrolysis peak intensities)/(sum of ertapenem related peak intensities) [9, 10]. Since both of the peaks of ertapenem and its hydrolysed, decarboxylated form may be observed in the mass spectrums of ertapenem solutions incubated with carbapenemase or non-carbapenemase producers, a cutoff value of logRQ was necessary to distinguish these two groups. The cutoff value was acquired based on receiver operating characteristic curve (ROC) which was conducted to determine the accuracy of the logRQ values to estimate the results of hydrolysis assay.

### Clinical blood cultures collection

Patient positive blood culture bottles which contained *Enterobacteriaceae* strains were collected from October 2015 to December 2016 in the clinical microbiology laboratory of Xinhua Hospital. Both of aerobic and anaerobic blood culture bottles (BD BACTEC, Bectom Dickinson) from different body parts of patients were included. After blood culture bottles were accepted at our laboratory, they were put in the BD BACTEC FX Blood Culture System (Bectom Dickinson) immediately until the positive signals were obtained. The blood cultures incubated in blood culture system for 5 days with no positive signals were considered as negative. The bacteria from blood cultures bottles with positive signals were collected under centrifugal force in serum separator tubes and were placed onto the target plate for directly identification by MALDI-TOF MS. If the bacteria were finally identified as *Enterobacteriaceae* strains, their hydrolytic activity against ertapenem were further performed by MALDI-TOF MS according to “MALDI-TOF MS based ertapenem hydrolysis assay” that mentioned above. *Klebsiella pneumoniae* ATCC 700603 provided by Shanghai Clinical Laboratory Center was included as the negative control, while one *Klebsiella pneumoniae* KPC-2 gene positive strain which identified before in our laboratory was included as the positive control. The conventional inoculation, identification (Microflex LT, Bruker Daltonics) and antimicrobial susceptibility test (Vitek 2 Compact, BioMérieux) of pathogens isolated from positive blood cultures were performed at the same time. Ertapenem, meropenem and imipenem were used as carbapenem agents in the research. The strains were identified as carbapenem-resistant *Enterobacteriaceae* (CRE) if they were resistant to at least one of the three carbapenem agents.

### Molecular assay

The carbapenemase genes, including *bla*_*KPC*_, *bla*_*IMP*_, *bla*_*NDM*_, *bla*_*VIM*_, *bla*_*GES*_ and *bla*_*OXA*-48_ were detected by Polymerase Chain Reaction (PCR) and DNA sequencing within the strains which were indentified as CRE [11].

## Results

### Strains for blood culture simulation

The strains collected here covered 7 species of *Enterobacteriaceae*, including *Klebsiella pneumoniae*, *Enterobacter cloacae*, *Enterobacter aerogenes*, *Escherichia coli*, *Citrobacter freundii*, *Klebsiella oxytoca* and *Raoultella ornithinolytica*. 32 carbapenemase producers contain 6 kinds of carbapenemases in total, including KPC-2, NDM-1, IMP-1, IMP-4, VIM-1 and VIM-2.

### Peaks of ertapenem and the hydrolysed, decarboxylated form

**Fig. 1:**
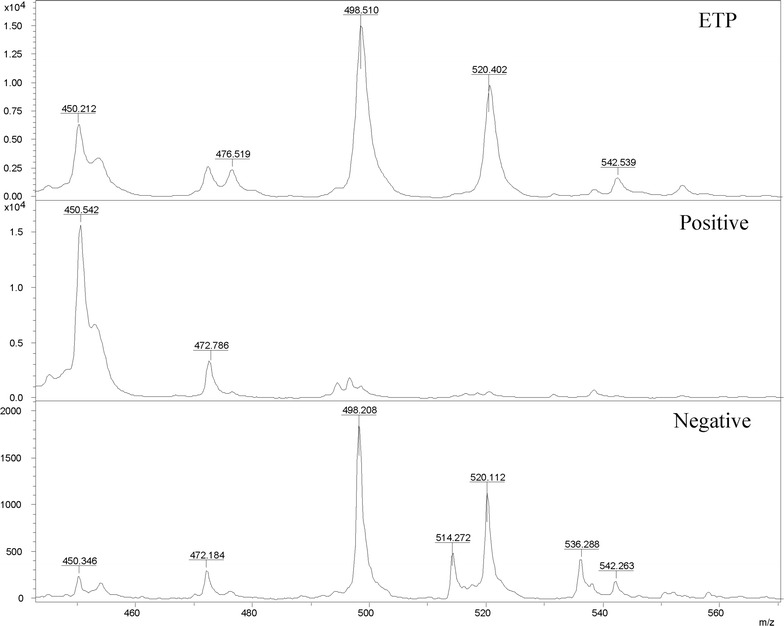
Peaks of ertapenem and their hydrolysed, decarboxylated form detected by MALDI-TOF MS. “ETP” means the peaks of ertapenem solution after 2 h incubation, “Positive” means the peaks of ertapenem solution after 2 h incubation with carbapenemase producers, “Negative” means the peaks of ertapenem solution after 2 h incubation with non-carbapenemase producers

Ertapenem intact peaks [M + H]^+^ at 476.5, [M + Na]^+^ at 498.5, [M + K]^+^ at 514.5, [M + 2Na]^+^ at 520.5, [M + Na + K]^+^ at 536.5 and [M + 3Na]^+^ at 542.5 and the peaks of their hydrolysed, decarboxylated form [M_hydr/decarb_ + H]^+^ at 450.5, [M_hydr/decarb_ + Na]^+^ at 472.5, [M_hydr/decarb_ + K]^+^ at 488.5, [M_hydr_ + H]^+^ at 494.5 and [M_hydr_ + 2Na]^+^ at 538.5 were all showed in Fig. 1. The potassium ion derived from human blood can combine with both of ertapenem and the hydrolysate and form peaks such as [M + K]^+^, [M_hydr/decarb_ + K]^+^, etc. These peaks were analyzed when mass spectrums of ertapenem solutions incubated with carbapenemase producers or non-carbapenemase producers were imported in Flexanalysis 3.0 software. Carbapenemase producers can be distinguished preliminarily by the following criteria: (1) almost absence of all ertapenem intact peaks; (2) presence of peaks corresponding to hydrolysed, decarboxylated form with high intensity.

### LogRQ value calculated with the intensity of peaks

**Fig. 2:**
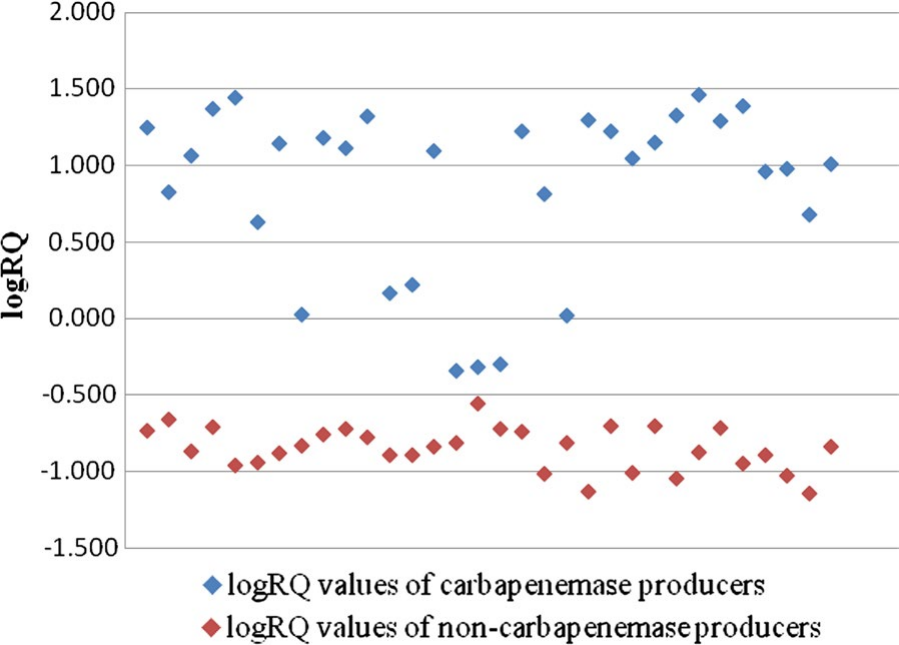
LogRQ values of 32 carbapenemase producers and 32 non-carbapenemase producers after 2 h incubation. LogRQ = log (sum of hydrolysis peak intensities)/(sum of ertapenem related peak intensities)

As a matter of fact, the subjective judgment of carbapenemase and non-carbapenemase producers according to the artificial observation of these peaks was ambiguous. We introduced logRQ value to analyze the intensities of these peaks. After 2 h incubation with ertapenem solution, the average of logRQ value of 32 non-carbapenemase producers were − 0.85 ± 0.14, and 32 carbapenemase producers were 0.87 ± 0.55 (Fig. 2).

### Cutoff value of logRQ

**Fig. 3:**
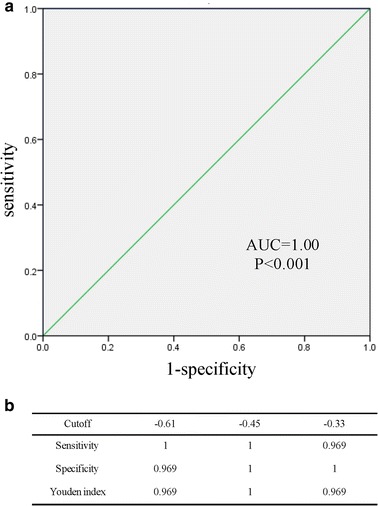
Statistical analysis with the logRQ values of the blood culture simulation. **a** ROC curves of the method that using logRQ values to identify carbapenemase producers. **b** Sensitivity, specificity and youden index with different cutoff values of logRQ

The receiver operating characteristic (ROC) curve of the method with logRQ values was drawn in Fig. 3. The ROC analysis results showed that the logRQ value was effective in distinguishing carbapenemase producers and noncarbapenemase producers (area under curve, AUC = 1, P value < 0.001). These two groups were discriminated by the cutoff value of logRQ − 0.45 with a sensitivity of 100% and specificity of 100%.

### Efficiency of MALDI-TOF MS based hydrolysis assay

A total of 385 clinical blood culture bottles contained *Enterobacteriaceae* strains from different body parts of 218 patients were collected. 81.3% (313/385) were sensitive to carbapenem, including *Escherichia coli* (207), *Klebsiella pneumoniae* (68), *Enterobacter cloacae* (15), *Klebsiella oxytoca* (6), *Serratia marcescens* (5), *Citrobacter freundii* (3), *Citrobacter koseri* (3), *Enterobacter aerogenes* (2), *Morganella morganii* (2), *Proteus mirabilis* (2). The logRQ values of all 313 isolates were < − 0.45 with the accuracy rate of 100%. And 18.7% (72/385) of clinical blood culture bottles contained CRE (Table 1). The logRQ values of 62 CRE were ≧ − 0.45, while the other 10 CRE were < − 0.45. Comparing with the detection of carbapenemase genes, MALDI-TOF MS based ertapenem hydrolysis assay of carbapenemase detection has a sensitivity of 92.5% (62/67) [95% CI 86.2–98.8%] and specificity of 100% (5/5) [95% CI 100–100%].

**Table 1:** The results of ETP hydrolysis assay and carbapenemase gene detection of CRE strains isolated from clinical blood cultures

Bacterial species (n)	Carbapenemase genes (n)	LogRQ value ≧ − 0.45	LogRQ value < − 0.45
*Klebsiella pneumoniae* (64)	KPC-2 (54)	54	0
	NDM-1 (5)	3	2
	Neg (5)	0	5
*Escherichia coli* (2)	NDM-1(2)	2	0
*Klebsiella oxytoca* (2)	NDM-1 (2)	2	0
*Enterobacter aerogenes* (2)	NDM-1 (2)	0	2
*Citrobacter freundii* (1)	KPC-2 and IMP-4^a^ (1)	1	0
*Morganella morganii* (1)	NDM-1 (1)	0	1
Total (72)		62	10

‘Neg’ means no carbapenemase genes detected

^a^The isolates that contained both *bla*_*KPC*-2_ and *bla*_*IMP*-4_ carbapenemase genes

## Discussion

MALDI-TOF MS has been used commonly for direct identification of microorganisms from positive blood cultures in clinical microbiology laboratories. There are various bacterial extraction methods for direct blood culture identification, including blood cells lysis [12–16], differential velocity centrifugation [17] and serum separator tubes usage [18]. Nowadays, MALDI-TOF MS has not only been used for identification of pathogens but also for detection of antimicrobial resistance [19, 20]. In our laboratory, serum separator tubes were used to collect bacterial pellets. After placing the pellets onto the target plate, MALDI-TOF MS was directly performed for identification. This protocol was routinely performed for positive blood cultures in our laboratory. The species identification reports of positive blood cultures usually delivered to clinicians within 30 min. In our previous research, the carbapenemase activity of *Enterobacteriaceae* strains that growed on Columbia blood agar was detected rapidly and accurately by MALDI-TOF MS based ertapenem hydrolysis assay. Based on that, the carbapenemase activity of *Enterobacteriaceae* strains in positive blood cultures collected from clinical patients was detected by the same assay. Different from the other researches, only simulated blood cultures were chosen here as the objects [9, 21, 22]. We first established a blood culture simulation method and calculated the cutoff values of logRQ. According to the logRQ values, we test the real positive blood cultures from patients to evaluate the efficiency of this method. The results revealed that this MALDI-TOF MS based ertapenem hydrolysis assay showed more clinical significance than before.

In our study, various species of *Enterobacteriaceae* strains which contained different types of carbapenemase genes were selected for positive blood culture simulations. These strains almost covered all the types of species and carbapenemase genes that could be detected in clinical microbiology laboratory. Therefore, MALDI-TOF MS based hydrolysis assay here is suitable to detect the carbapenemase activity of all species of the *Enterobacteriaceae* strains in blood cultures.

The limitation of MALDI-TOF MS based hydrolysis assay is that this method could only be used to detect carbapenemase activity which is one of the most important drug resistant mechanisms of CRE. In our study, 5 CRE strains were identified as non-carbapenemase producer by MALDI-TOF MS based hydrolysis assay. Besides that, no carbapenemase genes which mentioned in Materials and Methods were detected in these 5 isolates. The reason may be the presence of other carbapenem resistance mechanisms [23]. 41.7% (5/12) NDM-1 carbapenemase gene-positive *Enterobacteriaceae* isolates were distinguished as non-carbapenemase producer with the logRQ values of below − 0.45. However, as reported before, carbapenemase-producing *Enterobacteriaceae* isolates which carried NDM-1 can hydrolyse carbapenem completely after 2 h incubation [21, 22, 24–26]. Pure colonies other than strains collected from blood cultures were taken as research objects in most of these researches. Compared with pure colonies grown on blood agar, the amount of bacteria in pellets collected from positive blood cultures is not easily detected which will lead to the false-negative results. In our study, there were several clinical blood culture bottles which contained two species of *Enterobacteriaceae* strains. These strains in the same blood culture bottles which all showed sensitive to carbapenem were identified as non-carbapenemase producers by MALDI-TOF MS based hydrolysis assay. Besides, as long as there was one strain in the blood culture bottles showed resistant to carbapenem, all these two strains were identified as carbapenemase producers. The results showed that there were no needs to separate the multi-microorganisms in positive blood cultures when detected carbapenemase activity by MALDI-TOF MS. This method can help to evaluate the effectiveness of carbapenem treatment timely.

The antibiotic sensitivity reports were essential for clinical treatment of patients whose blood cultures were determined as positive. At our laboratory, MALDI-TOF MS has been routinely used to directly identify the species of strains from positive blood cultures. Our data showed that MALDI-TOF MS based ertapenem hydrolysis assay can directly detect carbapenemase of *Enterobacteriaceae* strains isolated from blood cultures with high sensitivity and specificity. Thus, when the assay is routinely performed for positive blood cultures, we could deliver the reports, especially positive results, to the clinicians within 3 h and provide them evidence to choose appropriate combined medication regimen for the patient early.

## Conclusions

MALDI-TOF MS based ertapenem hydrolysis assay is a rapid, accurate, effective and economic method to detect carbapenemase activity of *Enterobacteriaceae* strains isolated from positive blood cultures. With the high specificity and sensitivity, this method can be used as routine protocol to detect carbapenemase in clinical workflows. So clinicians can acquire meaningful reference early to treat bloodstream infection caused by *Enterobacteriaceae* strains.

## Abbreviations

MALDI-TOF MS: matrix-assisted laser desorption ionization time-of-flight mass spectrometry
CRE: carbapenemase-resistant *Enterobacteriaceae*
ETP: ertapenem
KPC: *Klebsiella pneumoniae* Carbapenemase
NDM: New-Delhi-Metallo-betalactamase
IMP: Imipenemase
VIM: verona metallo-β-lactamase
OXA: Oxacillinase
